# Additive manufacturing of NiTi shape memory alloy and its industrial applications

**DOI:** 10.1016/j.heliyon.2023.e23369

**Published:** 2023-12-06

**Authors:** Thywill Cephas Dzogbewu, Deon Johan de Beer

**Affiliations:** a Department of Mechanical and Mechatronics Engineering, Central University of Technology, Free State, Bloemfontein, South Africa; b Centre for Rapid Prototyping and Manufacturing, Central University of Technology, Free State, Bloemfontein, South Africa

**Keywords:** NiTi, Superelasticity, Shape memory, Actuation, Additive manufacturing, Laser powder bed fusion

## Abstract

NiTi shape memory alloys are the prime choice for many engineering and biomedical applications due to their unique response to environmental/external stimuli. The capability of laser powder bed fusion (LPBF) to additively manufacture high-quality density NiTi alloy with intricate geomaterial configuration, good surface quality, and chemical homogeneity makes the LPBF process the preferred choice among other additive manufacturing (AM) methods for manufacturing the NiTi alloy. The AM process parameters have a decisive effect on the functional and mechanical properties of NiTi alloy. There is a need to understand the resultant effect of the interrelationship between the process parameters on the final NiTi additive manufactured structures. The inherent high rate of melting and cooling of the LPBF process resulting in high internal stress could cause adverse effects such as cracks and Ni-loss which are detrimental to the phase transformation temperature of the NiTi alloy. Despite the current challenges, the literature reveals that LPBF NiTi components demonstrated functional and mechanical properties according to the ASTM standard and have been used widely for biomedical applications due to its stress-strain hysteresis, which is similar to bone tissues. The alloy is also used extensively for high-value engineering applications such as the automotive and aerospace industries due to its actuation properties.

## 1 Introduction

Shape memory alloys are smart alloys that have the ability to “remember” their original shape after being subjected to stimulus [1]. They are stimulus-sensitive alloys that can recover their original shape after deformation by heating or unloading. Although the ability of smart alloys to remember their original shape is generally termed as “shape memory effect”; technically when the recovery process is driven by temperature the resulting behaviour is termed “shape memory” and when it is driven by loading it is termed “superelasticity” [2]. Superelasticity (pseudoelasticity) is the ability of the material to sustain elongation or strain (3–8% for NiTi alloys) after deformation [3]. Apart from the shape memory effect and the superelasticity, shape memory alloys (**SMAs**) are also known to possess damping properties, high corrosion resistance, high resistance to oxidation, biocompatibility properties, etc. [1,2]. Due to these unique properties, they are used widely, especially for engineering and biomedical applications [4]. They have become the focus of many academic and industrial research because they are preferable as compared to other biomaterials [1] and engineering materials [5].

Since the discovery of SMAs in 1932 by Arne Olander [6] and the serendipitous identification of the unique shape memory properties of NiTi by Beuhler [7] in 1963 at the United States Naval Ordnance Laboratory and commercialized under the trade name Nitinol (an acronym for Nickel Titanium Naval Ordnance Laboratories), there has been increasing demand for SMAs for various industrial applications. There are three main categories of SMAs [1], namely: Cu-based, Fe-based, and NiTi-based shape memory alloys. Cu-based (eg. CuZnAl, CuAlNi) and Fe-based (eg. FeMnSi) SMAs are relatively low-cost and readily available commercially, as compared to NiTi-based shape memory alloys [8]. However, their poor thermal mechanical performance; low corrosion resistance; and poor stability limit their potential industrial applications [1]. NiTi and its alloys are known to have demonstrated high resistance to oxidation as compared to other SMAs; excellent damping characteristics; biocompatibility with unique functional stability, and has taken the centre-stage for industrial applications.

Despite the appealing properties of NiTi SMAs for many industrial applications, it is well documented that NiTi suffers from poor workability due to the high ductility, reactivity, and rebound effect, causing high tool wear during the manufacturing process [9], making it impossible to produce 3D structures with complex geometrical configuration via the conventional methods [10]. As a result, only structures of simple geometries such as tubes, rods, wires, and sheets are normally produced [11]. NiTi is conventionally produced as ingots via self-propagating high-temperature synthesis (SHS), vacuum arc remelting (VAR), or vacuum induction melting (VIM) processes [12]. The melting process is followed by hot working and tooling till the required shape of the object is obtained [13]. Obtaining a homogeneous NiTi alloy manufactured by the classical methods requires multiple re-melting of the ingot. The multiple melting can lead to carbon and or oxygen contamination of the alloy. The impurities would affect the microstructure of the NiTi alloy, hence the shape memory effect properties [12]. Elahinia et al. [12] touted that the method used to produce NiTi alloy is very crucial. The process of melting the metallic powder determines the quality, functionality, and behaviour of the NiTi alloy when in service. The literature reveals that the classical methods (SHS, VIM, and VAR processes) of producing the NiTi alloy lacks the capability to produce the alloy without contaminations and non-near-net-shape structures. To enhance the geometrical, technical, and functional efficiency of the NiTi alloy, an alternative manufacturing approach was initiated [1], as for an improved performance near-net-shape that would enhance the service efficiency of a component, an alternative method would be paramount [14]. Following from the needs identified, production of the NiTi SMA with intricate shapes (e.g. customized porous structures, back tapers, special lattices or hollow structures, and intricate cooling channels) makes AM an attractive alternative production route for the NiTi alloy. It is indicated that the AM processing routes could produce a homogenous microstructure without contamination due to the vacuum nature of the AM systems [15,16].

AM technology, which is considered a renaissance of the manufacturing industry [17], has become the prime choice technology to resolve the drawbacks imposed on the NiTi alloy by the classical manufacturing methods (SHS, VIM, and VAR processes) [4]. The AM manufacturing routes are a complete departure from the long-standing subtractive manufacturing paradigm that relies on material removal to produce desired geometries [18]. The AM strategy is a topology optimization process [19] that produce complex shapes monolithically, eliminating the numerous assembly steps of the conventional approach [20]. Due to the advantages offered by the AM process (production of customized light weight structures, eliminating of waste production, eliminating/reducing assembling steps) [21], there has been continuous research focussing on manufacturing the NiTi alloy via AM manufacturing routes to improve its geometrical, technical, and functional efficiency. As a result of the perceived advantages AM could add to NiTi SMAs, there has been intense research focus on manufacturing NiTi via AM routes and review publications on SMAs to understand the research status [1,4,22].

The recent review work of Wen et al. [4] focuses on the research status and prospect of AM NiTi SMAs; discussing the effect of some process parameters on the shape memory properties of NiTi alloys. Sabahi et al. [1] reviewed the various AM manufacturing routes for producing NiTi alloys, discussing its biomedical applications. Wang et al. [23] and Khoo et al. [24] also reviewed the effect of the AM manufacturing parameters on the various properties of AM NiTi samples. Various AM samples of NiTi were successfully produced for various academic investigations [25,26] and industrial applications [27,28]. Owing to the newness of AM technology and its rapid development via intense academic and industrial research resulting in high volumes research output, there is a need for frequent reviews to continually understand the current research status and how to improve the AM process for NiTi application. The current review seeks to highlight the current AM processes that are used to produce the NiTi alloy; its advantages, limitations, and how it could be improved. In addition, the effect of the principal AM process parameters on the shape memory effect would be discussed, including major engineering and biomedical applications.

## 2 Methodology

A general electronic databased search was conducted to access relevant published documents on AM of SMAs. From the initial output, high-cited index articles directly relating to the topic were carefully reviewed and from the snowball principles [29] similar relevant articles from well-known authors were also identified. From the initial review of the outstanding cited index articles, the search terms were refined to access articles on AM of NiTi alloys. The main refined search terms that were used to investigate how successfully AM processes have been used to produce NiTi alloy, its industrial applications, and the current challenges are: *NiTi, shape memory alloys, superelasticity, pseudoelasticity, 3D printing, additive manufacturing (AM), selective laser sintering (SLS), direct metal laser sintering (DMLS), direct selective laser melting (DSLM), selective laser melting (SLM), powder bed fusion (PBF), laser powder bed fusion (LPBF), electron beam melting (EBM), directed energy deposition (DED), wire arc additive manufacturing (WAAM), conventional manufacturing (CV), biomedical applications, engineering applications, and data preparation software.* To be able to access relevant articles that meet multiple criteria due to the multidisciplinary nature of the topic, Boolean search operators were used combining the search terms (eg. “NiTi” AND “additive manufacturing” OR ″3D printing” OR “Rapid prototyping” OR “Laser powder bed fusion” OR “Electron beam melting” OR “Selective laser melting” OR “Direct metal laser sintering” OR “Directed energy deposition” OR “Wire arc additive manufacturing” AND “Shape memory alloys” OR “Superelasticity” OR “Pseudoelasticity” AND “Biomedical applications” OR “Engineering applications” OR “Data preparation software”).

The combination of the search terms made it possible to identify published articles that present the current state of using AM to produce NiTi alloys.

**Fig. 1:**
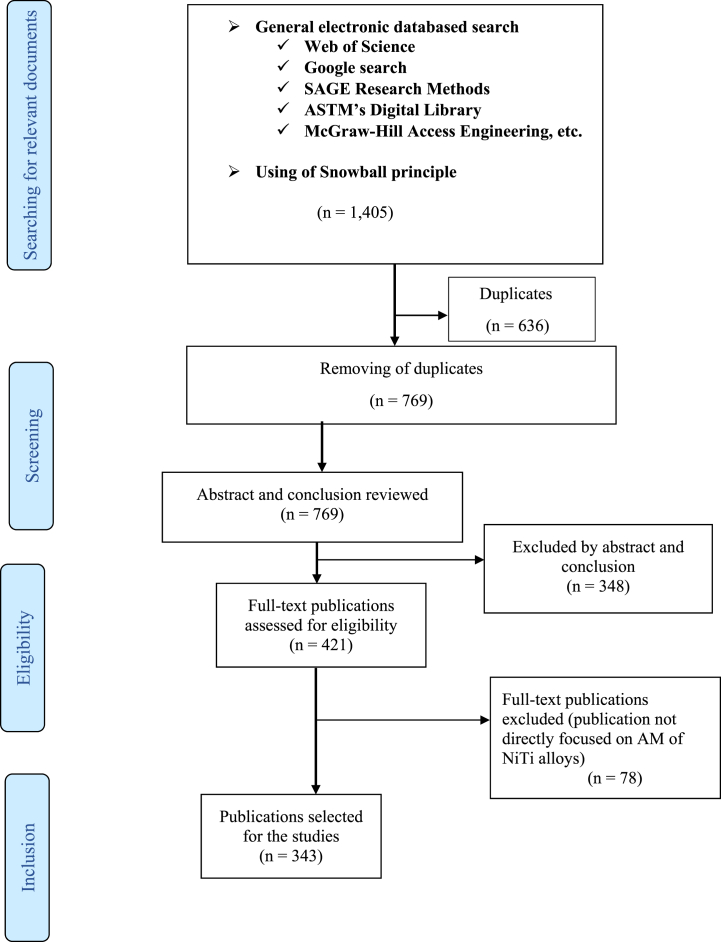
Step-by-step exclusion and inclusion criteria of selectin relevant published articles.

The articles were selected based on the content of the published documents and their relevance to the current topic. Only peer-reviewed documents were considered to ensure that every piece of information included in the current analysis has some degree of scientific credibility and reliability. Other documents that cannot be scientifically substantiated were not considered. The inclusion and exclusion criteria adopted for selecting only relevant published articles for the study is presented in Fig. 1. A step-by-step screening/eligibility approach was adopted to ensure only relevant articles that will contribute to the study were included in the final selection. The step-by-step selection approach make it possible that every document is carefully scrutinized to ensure only verified information are included in the current study. This systematic review approach provides a wholistic view of the current state of AM process capability to produce NiTi alloys and their industrial applications.

The inclusion/exclusion criteria for searching the various databases were specified to locate any relevant article that was published since the inception of rapid prototyping in the 1980s till the present day (2023), written in English language and focused on AM methods of NiTi alloys and their applications. The selection process commenced with reviewing the abstracts and conclusions of the documents outputs from the various databases. A preliminary screening of the content and quality of the published documents was conducted. Articles whose content did not focus mainly on the use of AM to produce NiTi alloys and their industrial applications were rejected. At the end of the screening process, a total of 343 verified scientifically verified documents were selected for inclusion in the current manuscript to examine the capability of AM methods to produce NiTi alloy according to the standard industrial qualifications, elucidating the current challenges and the way forward.

Although a deliberate attempt was made to search for every available electronic document on the internet about AM methods used to produce NiTi alloys and their industrial applications, other non-electronic documents that might contain relevant information could not be included in the current review.

## 3 Additive manufacturing of NiTi SMAs

There are AM technologies and metal AM has been thriving since the 1990s [30]. This could be related to the structural integrity offered by metallic products. Metallic materials generally demonstrated higher load-bearing capacities as compared to other materials such as polymers [31]. Metal AM (MAM) technologies rely on metallic feedstock in form of wires, and powders [32] to print 3D structures of intricate geometries. The MAM technologies are classified based on the type of feedstock and energy source used to melt/sinter the metal in sequential layers. The two main sources of energy used by MAM systems are laser beams and electron beams [30]. Most of the MAM systems use laser (SLS, SLM, DMLS) to print the 3D structures layer-layer while others use electron beam (EBM). For the purpose of universal identification, ISO/ASTM 52900:2021 [33] classified the AM systems into seven categories (powder bed fusion-PBF, sheet lamination - SL, directed energy deposition – DED, material extrusion - ME, vat photopolymerization - VP, material jetting – MJ and binder jetting - BJ), based on their operational mechanisms. PBF, SL, and DED are the main AM mechanisms according to the classification of ISO/ASTM 52900:2021 that use metallic feedstocks. PBF AM technologies are mainly used to produce intricate 3D structures of high resolution and rigorous build geometrical accuracy [34], while SL AM technologies are used to join different metallic objects to produce 3D structures with specific properties [35], and DED is normally used to repair/refurbish metal components [36]. PBF AM technologies emerged as the technology of choice for producing intricate 3D structures with tailored geomaterial configurations [16,37]. The PBF technology is generally classified into two categories, namely electron beam melting (EBM) technology and laser powder bed fusion (LPBF) technology [10]. The EBM technology has been used to produce NiTi alloy successfully. Otubo et al. [38], confirms that the EBM technology could be used to produce NiTi with a homogenous microstructure without carbon and oxygen contamination as compared to the conventional methods of manufacturing. Recent publications [39,40] also indicated that NiTi 3D structures fabricated by EBM demonstrated shape memory and superelastic properties as compared to those manufactured by conventional methods.

**Fig. 2:**
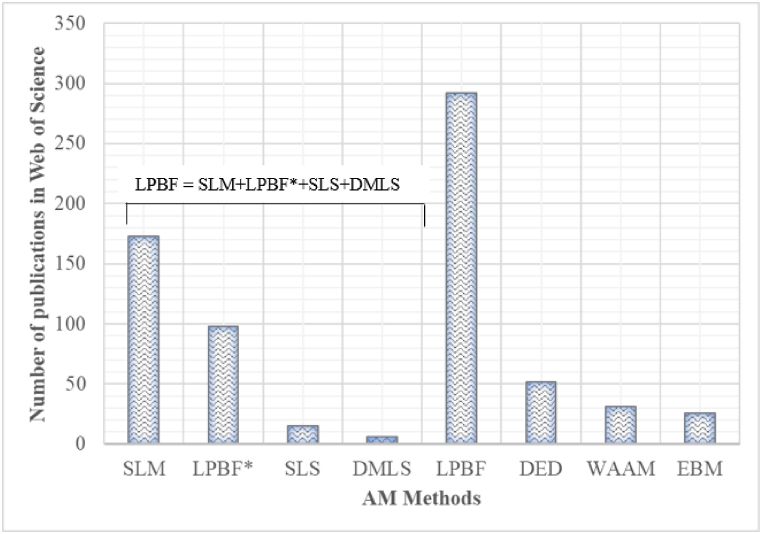
AM methods used to manufacture NiTi alloys and corresponding number of publications in Web of Science database. (LPBF = LPBF* + SLM + SLS + DMLS). LPBF* refers to publications that the author/s uses the LPBF terminology to describe the manufacturing process).

**Fig. 3:**
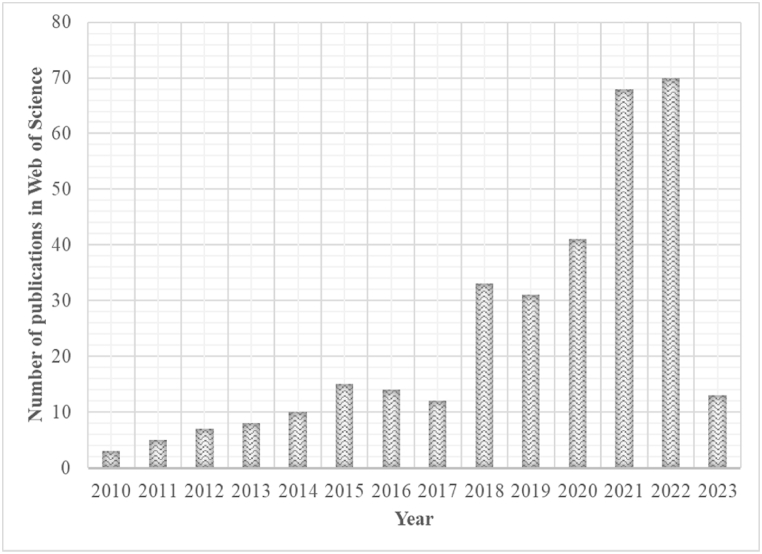
Number of annual AM NiTi alloy publication outputs in Web of Science database.

A thorough review of the literature was conducted in the Web of Science database to identify AM technologies that are normally used to produce NiTi shape memory alloys (Fig. 2). It was revealed that SLM - a subset of the laser powder bed fusion (LPBF) method - is most used for manufacturing NiTi SMAs for various industrial applications, which is also evident through the number of publications on NiTi alloy that continues to increase (Fig. 3).

(Note: The search in Web of Science database was conducted on the 23^rd^ of February 2023. The number of publications for 2023 is expected to be more than that of 2022 based on the current trend).

The LPBF technology might be preferred to the EBM due to the superior manufacturing capabilities of the LPBF technology [4]. It is documented that the EBM manufacturing technology lacks the ability to produce 3D structures with geometrical precision due to the large beam size [41], which results in the production of a large molten pool [42]. As a result, it is difficult to use EBM to produce intricate geometries according to the CAD (computer-aided design) file. The EBM process required provision for large machining allowances to permit extensive post-processing activities to reach the required geometry, according to the CAD design and surface quality. The herculean post-processing task would lead to increase in production cost, time and material wastage [41]. The undesirable extensive post-processing step of the EBM-manufactured components undermines the monolithic manufacturing advantage purported by the AM manufacturing process [18]. However, the current market demand seeks production of intricate 3D NiTi structures with tailored geometrical configurations, since the final NiTi AM build components are normally used for high-value industrial applications (health, automobile, aerospace, etc.) that require high-quality surface finish products with precise geometrical dimensions [43]. It could be postulated that due to the inherent limitations of EBM, the LPBF technology becomes the preferred method for production NiTi shape memory alloys for various industrial applications. From Fig. 2 it is confirmed that less is reported in the literature about other MAM processes, in favour of LPBF methods [2]. Although WAAM is the second most used AM manufacturing method in Web of Science data-based for NiTi alloy, it is less in dimensional precise as compared to EBM, DED, and LPBF, due to the wire type feedstock method [44]. WAAM samples demonstrate less geometrical accuracy and quality as compared to LPBF, EBM, and DED samples [44,45].

**Fig. 4:**
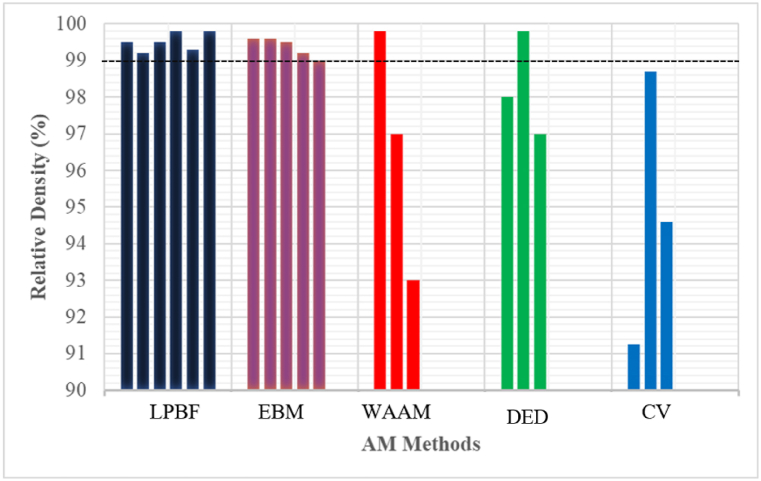
Densification of NiTi SMAs produced via different AM and CV methods. (The red dotted line represents good densification). (Data source: LPBF - [68,[72], [73], [74], [75]]; EBM - [39,40]; WAAM - [76]; DED - [77,78]; CV - [79,80]). (For interpretation of the references to colour in this figure legend, the reader is referred to the Web version of this article.)

Wang et al. [46], reported that the LPBF technology could be used to produce intricate structures with high resolution and rigorous build accuracy with a dimension error lower than 100 μm. Xiong et al. [47], produced 3D NiTi samples of 52 μm in size with <2 μm surface roughness, which might be practically impossible to produce using EBM and other AM manufacturing processes. The shape memory and superelasticity properties of the LPBF NiTi thin walls were better than the samples manufactured using conventional methods. The experimental results of Wang et al. [26], and Xue et al. [48], also demonstrated that the shape memory and superelasticity properties of NiTi components manufactured by LPBF were preferable as compared to other AM processes. The results reported by other non-LPBF technology that were used to produce NiTi components (Fig. 4, Fig. 8) were not preferable to 3D components manufactured using LPBF technologies. The outstanding manufacturing capability of the LPBF technology to spawn 3D components with geometrical precision, preferable shape memory and superelastic properties, might be the reason why the research community has focused on using the LPBF process to study the NiTi shape memory alloys. The current review would proceed to investigate how the principal process parameters of LPBF affect the shape memory and superelastic properties of NiTi including the challenges of using the LPBF process to produce the NiTi alloys and the main industrial applications.

**Fig. 5:**
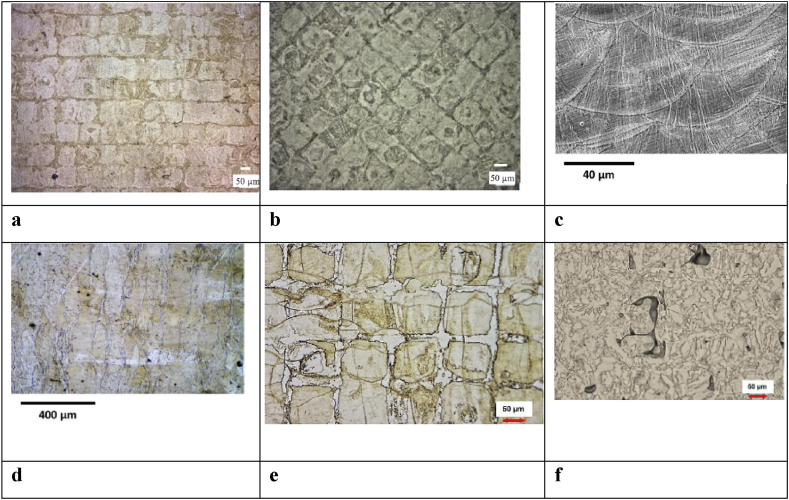
a) LPBF NiTi SMA sample built with alternating x/y scanning strategy in the longitudinal direction at 250 W [11], b) LPBF NiTi SMA sample built with alternating ± 45° to in the vertical orientation at 250 W [11], c) Longitudinal view of LPBF NiTi SMA sample built at 150 W [75], d) Perpendicular view of LPBF NiTi SMA sample built at 150 W [75], e) Perpendicular view of LPBF NiTi SMA sample built at 250 W [62], f) Perpendicular view of LPBF NiTi SMA sample built at 100 W (un -optimum process parameter) [62].

**Fig. 6:**
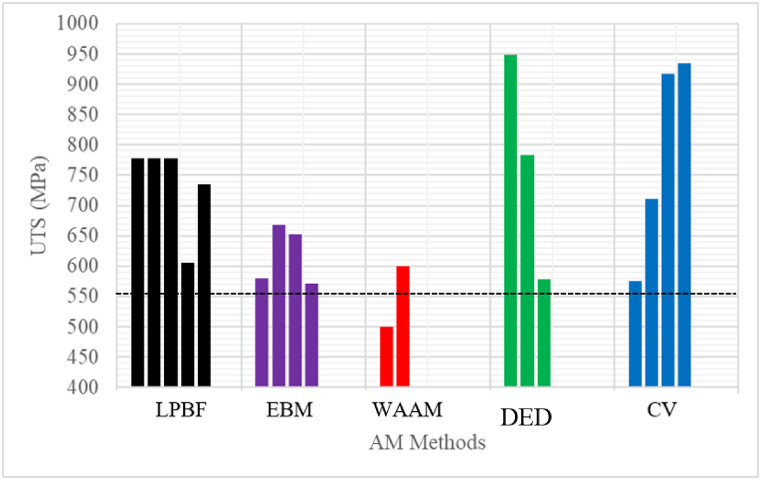
UTS of AM samples and CV samples. (The red dotted line represents ASTM required standard. (Data source: LPBF - [11,72,74,96,97]; WAAM - [45,[98], [99], [100]]; EBM - [39,101]; DED - [77,102]; CV - [11,72,103]). (For interpretation of the references to colour in this figure legend, the reader is referred to the Web version of this article.)

**Fig. 7:**
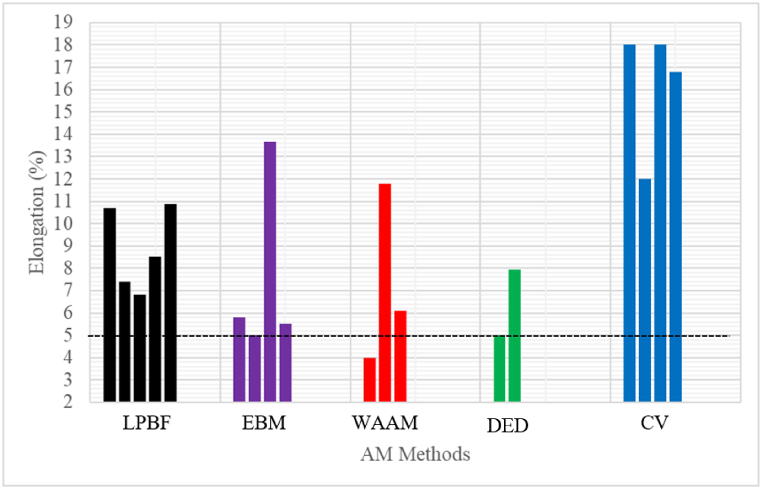
Percentage elongation of AM samples and CV samples. (The red dotted line represents ASTM required standard. (Data source: LPBF - [11,72,74,96,97]; WAAM - [45,[98], [99], [100]]; EBM - [39,101,104]; DED- [78,105]; CV - [11,72,78,103]). (For interpretation of the references to colour in this figure legend, the reader is referred to the Web version of this article.)

**Fig. 8:**
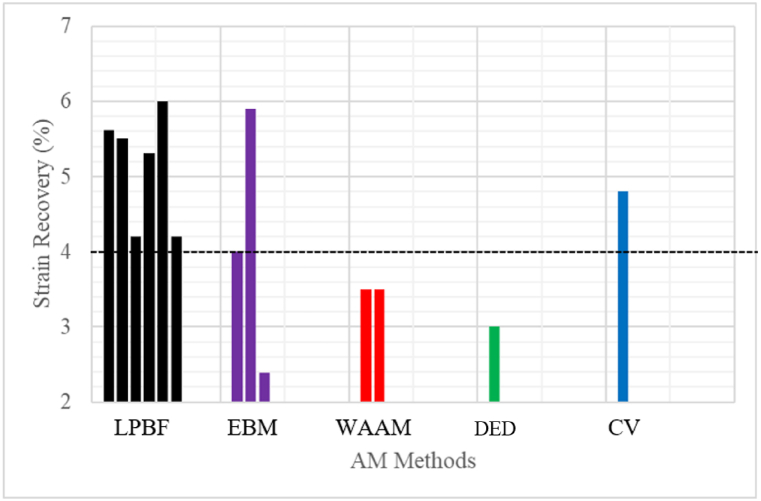
Strain recovery of AM samples and CV samples. (The red dotted line represents the required ASTM standard). Note: All the values presented are first-cycle results. (Data source: LPBF - [48,57,63,63,111]; WAAM - [99,100,112]; EBM- [40,101]; DED - [113]; CV – [103]). (For interpretation of the references to colour in this figure legend, the reader is referred to the Web version of this article.)

## 4 Laser powder bed fusion of NiTi SMAs

SMAs exhibit austinite phase (parent or original phase) at high temperatures and martensitic phase when cooling below the transformation temperature [49]. The martensitic phase is self-accommodating (a phase with many sheared platelets that slip very readily allowing twinning deformation on the interface of the platelets that can accommodate maximum elongation in the direction of the applied force) with many variants rendering the deformation in the martensitic phase zero [50]. At temperatures above the transformation temperature, the deformed structures of the martensite phase (the twinning platelets) revert to the single orientation of the austinite phase, and the material is said to regain its original shape [49,50]. This crystallographically reversible martensitic transformation, which is dependent on the transformation temperature, is a function of the alloy composition and manufacturing routes. Therefore, it is very important to examine the effect of the principal process parameters (laser power, scanning speed, powder layer thickness, and hatch distance) of the LPBF process on the shape memory effect and the superelasticity properties of NiTi SMAs. The principal process parameters of the LPBF process have a direct decisive effect on the evolution of the microstructure, mechanical properties, functional properties, surface quality and phase transformation behaviour of the NiTi SMAs. The principal process parameters determined the amount of laser energy density delivered onto the powder bed to melt the NiTi powder particles. The laser energy density influences the shape memory effect (SME) (response to thermal stimuli) and superelasticity (response to mechanical stimuli). The laser energy density is normally expressed as:

$$E=\frac{P}{vht}$$

where E − Energy input (J/mm3), P – laser power (W); v – scanning velocity (mm/s); h – hatch spacing (mm); and t – powder layer thickness (mm) [51].

It is widely reported that the martensitic transformation temperature monotonously varies with the individual changing of the process parameters. The variation of any of the principal process parameters variable changes the martensitic transformation temperature. In-depth knowledge about how the changing of the process parameters affects the transformation temperatures of NiTi gives the manufacturing engineer the ability to produce NiTi intricate samples with tailored functional properties. The martensitic transformation temperatures of NiTi alloy generally increase with increasing energy density of the powder bed [52]. Shi et al. [53] developed a quadratic regression model to determine the effect of laser power and scanning speeds on the NiTi transformation temperatures and reported that the martensite start temperature and austinite finished temperatures increase with increasing scanning speeds and decrease with decreasing laser powers. Their investigation reveals that the functional and mechanical properties of SMA alloy could vary at the same laser energy density. Therefore, it could be concluded that the variation of the laser powers and scanning speeds should be the focus of determining optimum process parameters, not the laser energy density.

The experimental investigations of Dadbakhsh [54] lead to the conclusion that high processing parameters (laser power 250 W, scanning speed 1100 mm/s) promote superelasticity of NiTi alloy while low processing parameters (laser power 40 W, scanning speeds 160 mm/s) increase the martensitic transformation temperature of the NiTi alloys. Wang et al. [55] reported that the martensitic transformation temperature decreases with increasing scanning speeds and increases with increasing laser powers, which is contrary to the experimental results of Shi et al. [53]. Bormann [56] noted that scanning speeds mainly have effects on the transformation temperature whereas laser power majorly influences microstructure (texture, crystallographic orientation, crystallite shapes and arrangement, and grain size distribution). It is also indicated that transformation temperature decreases with increasing hatch distance [55]. Moghaddam et al. [57] also stated that hatch distance is a very effective process parameter that could be used to tailor the size, shape, and orientation of grains. Hence the superelasticity properties of NiTi could be modulated by varying the hatch distance during the LPBF process of NiTi alloy. The properties of the NiTi alloys could be controlled by adapting the Ni_4_Ti_3_ precipitates via the change in the laser energy density. It is also known that the thermomechanical properties of NiTi alloys produced with the same energy density could yield different results making it difficult to ensure consistency and repeatability of obtaining NiTi with consistent mechanical properties at the same energy density [58].

Composition analysis by Shi et al. [53], reveals that the variation normally observed in the transition temperature of as-built samples at the same energy density is as a result of Ni-loss in the building process [56,59]. Different amounts of Ni could be lost at the same energy density based on the selected laser power and scanning speed. The ratio of the elements (Ni/Ti composition ratio) affects the phase transition temperature of NiTi SMAs. It is important to optimize the LPBF process parameters to avoid evaporation of Ni to maintain the right composition of the Ni/Ti ratio for precise control of the phase transformation temperature. The ability of the manufacturing engineer to control the amount of Ni loss in the final built components could provide the window of precise controlling of the transformation temperature of the alloy. The era of controlling the Ni-loss during the LPBF process would pave the way for producing novel NiTi smart structures with pre-determined transformation temperatures and thermomechanical properties. Such NiTi samples manufactured by LPBF process with precises pre-determined NiTi composition would demonstrate 4D printing characteristics [60] as designed by the manufacturing engineer. 4D printed objects are 3D printed objects with smart materials that have the capability to respond to an external stimulus (heat or load). The external stimuli could cause the shape or physical properties of the material to change over time [61]. Due to the huge advantage offered by 4D printed objects, there is ongoing intense industrial and academic research of using the LPBF process to produce NiTi 4D printed samples [25,62,63].

### 4.1 Densification

Densification of AM fabricated parts depends on the process parameters. The principal process parameters determined the densification of a built component. Since the current analytical software are not able to predict the exact optimum process parameter values, researchers have adopted a time and energy-consuming trial-and-error approach to determine optimum process parameter windows [19,64]. Optimum process parameters would produce a molten pool with a complete molten powder particle that could ensure good densification of the solidified tracks; layers and the resultant densified 3D components. Non-optimum process parameters are the main causes of defects such as cracks leading to low densification. Due to the large number of process parameters (machine-based input parameters, material-based input parameters, process input parameters, etc.) [31], that affect the determination of an optimum energy density, a varied range of process parameter windows has been reported. Haberland et al. [65], reported 200 J/mm^3^ and Meier et al. [66] reported 85 J/mm^3^ as the energy density required to produce dense samples. The degree of dispersed results presented in the literature for optimum energy density attests that the AM processes have not fully matured, although industrial applications are already evident [18,67]. As explained by Zhu et al. [68], predicting the optimum energy density for producing a 3D sample is very challenging. Low densification can occur at an energy density below or above the critical energy density [69]. A low energy density below the critical value would not be sufficient to melt the NiTi metallic powders. A high energy density above the critical value would increase the volume of the molten pool [70], which could entrap gas bubbles and prevent them from diffusing to the surface of the molten pool. The entrapped gas bubbles within the solidified samples would increase the porosity of the samples. It is stated that high energy levels above the critical value could trigger balling effect and void formation [69]. Haberland et al. [65], and Walker et al. [71], reported decrease of relative density at extremely high laser energy density. The study of Saedi [62], established that to produce a dense NiTi component a combination of high laser power (250 W) with a high scanning speed (1000 mm/s) or low laser power (100 W) with a low scanning speed (125 mm/s) should be used. The authors noted that the low laser power samples demonstrated preferable superelasticity properties as compared to the high laser power samples.

The literature defined good densification at a threshold of 99 % relative density [68,81]. From Fig. 4, LPBF, EBM, and some WAAM and DED manufactured samples have demonstrated good densification (black dotted line). This is evident that with optimum process parameters, AM manufactured samples could demonstrate good densification. The densification-levels demonstrated by the LPBF samples might be one of the reasons why the research community and industry practitioners have focused on using LPBF for NiTi samples with tailored geometrical configurations. It is generally difficult to obtain 100 % densification, especially when the starting material is elemental powder (Ni and Ti) [32,82]. The low densification demonstrated by CV samples indicated that the AM methods are generally superior (Fig. 4).

### 4.2 Microstructure and precipitates of NiTi LPBF samples

During the LPBF process, the NiTi powder particles at the top layers undergo a rapid melting-solidification process while the solidified layers beneath experience thermal cycling. The unique thermal cycle based on the selected processing parameters produced NiTi-built components with complex microstructural evolution, which influences the transformation temperatures and thermomechanical properties [58]. It is also reported that the variation in the transformation temperature could be triggered by precipitates or dislocations [77,83,84]. However, since almost all LPBF NiTi samples undergo post-heat treatment, it is expected that the heat treatment is supposed to homogenize the microstructure and dissolve/remove precipitates and reduces dislocation density, internal stress, melt un-melted particles, and other minor defects as a result of the LPBF manufacturing mechanism. On the contrary, the experimental report of Speirs et al. [85], and [59] Wang et al., indicated that heat treatment temperatures (1273 K for 2 h) (*solution annealing*) could not change the transformation temperature of the heat treated samples with the non-heat treated counterparts. Only the “transformation interval” - thus the width of the transformation peaks - was reduced because of the heat treatment. It could therefore be inferred that the presence of dislocations, internal stress, precipitates, un-melted powder particles, and defects due to the LPBF building mechanism may not be the principal contributors to the variation in the transformation temperatures, but rather the principal processing parameters are responsible for the change in the transformational temperatures. This inconclusive observation of heat treatment not having a decisive effect on the transformational temperatures (functional properties) could probably be motivated by the manufacturing mechanism adopted by the LPBF process. The thermal recycling process experienced during the building process is similar to heat treatment (aging or solution annealing), which could cause the precipitation of Ni_4_Ti_3_ particles. Although heat treatment could dissolve the Ni_4_Ti_3_ precipitates, reduce chemical and microstructural inhomogeneities, dislocation density, and residual stresses [86], other precipitates are also induced during the heat treatment (Ni_3_Ti_2_, and Ni_3_Ti), [87]. The presence of these precipitates in the as-built and heat-treated samples seems to minimize the effect of heat treatment on the phase transformation temperatures of the alloy. On the contrary, other research outputs [[88], [89], [90]] have indicated that the superelasticity and shape memory behaviours of NiTi are highly dependent on aging temperature, aging time, and cooling rate. Saedi et al. [87], demonstrated that during heat treatment the formation of precipitates (Ni_4_Ti_3,_ Ti_2_Ni, TiNi_3_, Ni_3_Ti_2_, and Ni_3_Ti) improves the phase transformation temperature of heat-treated LPBF samples as compared to the as-built samples. In the view of Haberland et al. [65], finely dispersed precipitates such as Ni_4_Ti_3_ increase the superelasticity behaviour of NiTi alloy, due to their ability to act as nucleation sites for martensitic transformation. However, coarsen precipitates, which are normally obtained after prolonged aging, can become detrimental by embrittling the metal matrix, which could reduce the superelasticity properties of the NiTi alloy.

Heat treatment also dissolved Ni-rich second phases in the austenitic matrix, thereby increasing the nickel content in the NiTi alloy. The increase in the nickel content could affect the transformational temperature by decreasing the martensitic start temperature [86].

It is also worth noting that the starting microstructure of LPBF NiTi samples are completely different (Fig. 5), due to the different process parameters and scanning strategies. Since the starting microstructures of LPBF NiTi samples are vastly unidentical, standard heat treatments might not necessarily produce the required expected microstructural result. Tailoring heat treatment strategies such as; holding time, and varying cooling rate among others, could enable the production of expected microstructures that could yield preferable shape memory and superelasticity results.

### 4.3 Ultimate tensile strength and percentage elongation (Mechanical properties)

Ultimate tensile strength (UTS) and percentage elongation give information about the load-bearing capacity of material before fracture. The UTS and elongation of as-built NiTi samples produced by the various AM manufacturing methods and CV in the literature are presented in Fig. 6. For medical applications, which is one of the major uses of NiTi alloy, the ASTM standard required that the UTS should not be below 551 MPa (Fig. 6: The black dotted line) and the percentage elongation should not be below 5 % (Fig. 7: The black dotted line) [[91], [92], [93]], and [94]. The current litterature demonstrates that all the as-built AM samples and heat-treated samples demonstrate the required minimum percentage elongation required for medical applications. Lu et al. [95], reported ultra-high ductility of 22.41% elongation for as-built LPBF samples. This particular result reveals that it is possible to produce LPBF samples with good ductility without any heat treatment, which supports the ultimate aim of producing AM samples monolithically in one manufacturing cycle without major post-manufacturing processes. For medical applications, it could be argued that the current level of maturity of the AM technologies to produce NiTi products according to the required ASTM standard is achievable. However, the superior manufacturing strategy of the LPBF process of producing 3D structures with rigorous dimensional accuracy make it preferable. The UTS and ductility of the CV samples are generally higher than the AM samples (Fig. 6, Fig. 7). This could be attributed to the high melting and cooling rate manufacturing process of the AM methods [31]. However, the inherent limitations (inhomogeneity, simple non-near net shapes) of the CV methods [10,14] have rendered them not to be the prime choice. The literature did not present adequate data on the ductility of DED, but it is reckoned that it should demonstrate a similar percentage of elongations as the other AM processing methods.

Initial research on the mechanical properties of AM NiTi samples were conducted under compression mode, because the initial AM NiTi as-built components have scarce processability and parts without a significant amount of porosity and defects [23]. The defect observed in the initial AM NiTi samples were detrimental to tensile properties. However, the optimization of the processing parameters and the current stage of maturity of the LPBF process has enabled the current researchers to produce NiTi-dense samples for testing in the tensile mode [106,107]. This is because a high number of NiTi devices for industrial applications operate under tension or partial tension conditions. Excellent reports were presented about the mechanical behaviour of AM NiTi alloys under compression mode. However, under tensile conditions, it is reported that AM NiTi alloys demonstrate low ductility (Fig. 7) as compared to the conventional samples. Some studies have even proven that conventional samples could demonstrate exceptional ductility of 30 % [108]. Several studies have also been conducted to increase the ductility of LPBF NiTi as-built components without heat treatment that yields good results [95,109,110].

### 4.4 Strain recovery and recovery ratio (Superelasticity and shape memory effect)

Current results show that the superelasticity and shape memory results of as-built LPBF samples of NiTi and heat-treated samples could have similar results. Moghaddam et al. [57] prove this by producing LPBF NiTi samples with optimum processing parameters of and reported a 5.62% tensile strain recovery and 98% recovery ratio without heat treatment. The authors reported that such results were not possible without heat treatment in the previous studies. Their contribution indicated the possibility of manufacturing LPBF NiTi components for patient-specific biomedical implants without heat treatment. Some studies reported a 100% recovery ratio for as-built AM NiTi samples and heat-treated samples at an initial strain of 4–5% [45].

From the plotted data in Fig. 8, the LPBF results seem to be preferable as compared to the other AM manufacturing process. The attention of most researchers is drawn to optimizing the process parameters of the LPBF process, and as a result, not much data is made available on the other AM process. For medical applications, the ASTM standard requires a strain value, not below 4% [78], - (Fig. 8: The black dotted line). The LPBF demonstrate a higher recovery strain as compared to other AM process, which could be due to the continuous improvement in the LPBF process parameters.

Both tensile strain and compressive strain recovery results did not provide a consensus view about the testing approach that yield a better strain recovery result. However, it is proven that building direction has a significant effect on the tensile strain recovery results. Moghaddam et al. [11], produced LPBF NiTi tensile samples in three different orientations: vertical, horizontal and edge orientations in x/y and ±45° alternating to the x-axis), (Fig. 5). The analysis reveals that the build direction and scanning strategies have a decisive effect on the microstructure, texture, ductility, functional stability, shape memory effect, superelasticity, and failure stress. The samples in the horizontal direction with x/y alternating scanning demonstrated a better superelasticity and shape memory effect as comped to samples that were printed vertically with ±45° alternating scanning strategy.

**Fig. 9:**
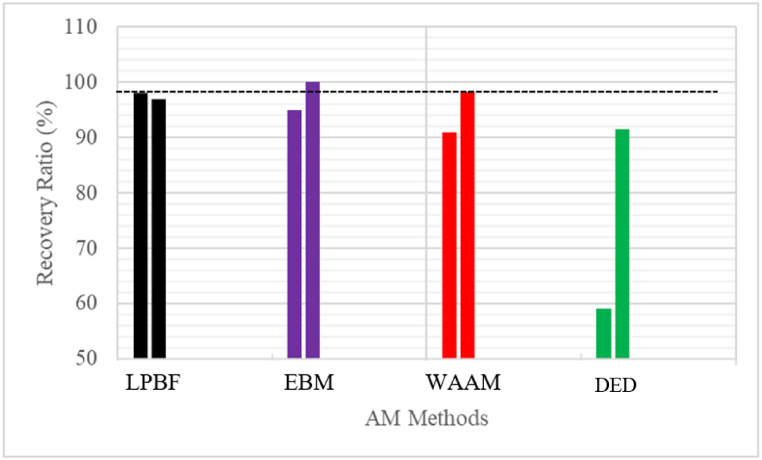
Recovery ratio of AM samples. (The red dotted line represents a good recovery ratio). Note: All the values presented are first-cycle results. (Data source: LPBF- [57,72]; WAAM- [45,98], EBM- [39,40], DED- [102,105]). (For interpretation of the references to colour in this figure legend, the reader is referred to the Web version of this article.)

In the view of McClung et al. [114], a good strain recovery ratio is close to 100 %. Moghaddam et al. [57] consider a strain recovery ratio of 98 % as a remarkable strain recovery ratio (Fig. 9: The black dotted line). The strain recovery ratio demonstrated that the LPBF samples could be considered good strain recovery values since they are close to 100 %. Khismatullin et al. [45], use the WAAM manufacturing method and reported a strain recovery ratio of 100 % for both as-built samples and heat-treated samples. These unique results attest that it is possible to produce AM samples with the maximum required recovery ratio without heat treatment. Notwithstanding, several research groups have focused their attention on improving the superelasticity properties of LPBF NiTi through post-heat treatment. Although the authors could not find any available data on the recovery ratio of CV samples, Moghaddam et al. [57] indicated that full strain recovery in as-cast NiTi samples is very rare.

## 5 Industrial application of additive manufactured NiTi 3D structures

**Fig. 10:**
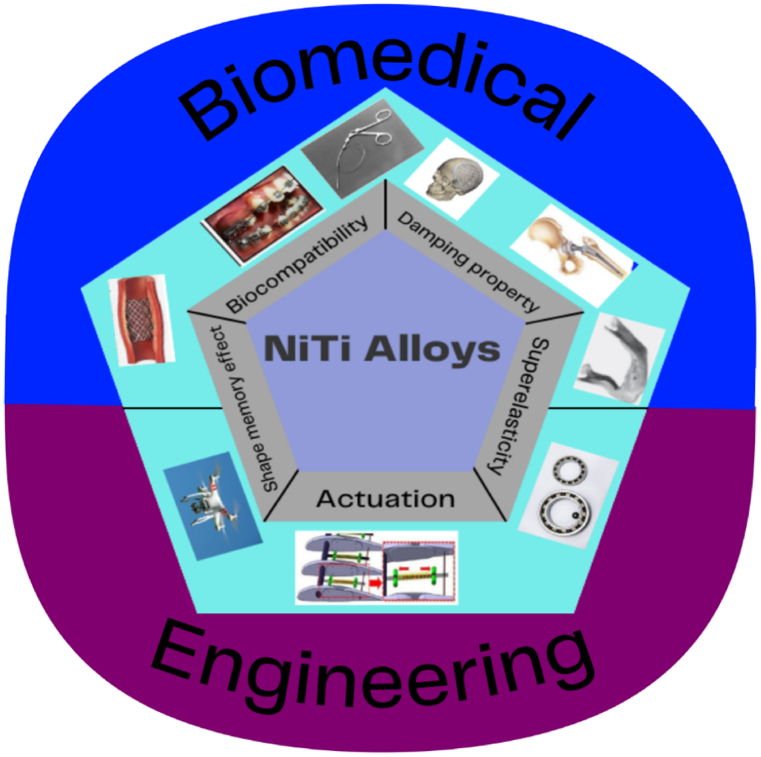
Shape memory properties and industrial applications of NiTi alloys.

The superelasticity and shape memory properties of NiTi have made them the prime choice for many industrial applications. NiTi devices are used for biomedical applications (Fig. 10) such as stents, surgical devices, etc. [28,90,[115], [116], [117]], and engineering applications (Fig. 10) such as aerospace [117,118], micro-electromechanical systems [28], actuators [28,115], couplings and fasteners [119], electrical safety devices [28], sporting equipment [117], etc.

### 5.1 Biomedical applications

**Fig. 11:**
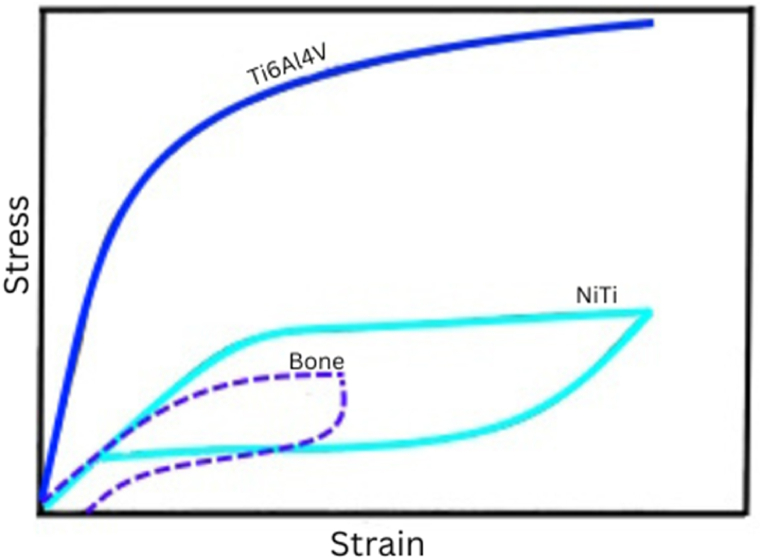
Schematic representation of stress-strain curve of Bone tissue, NiTi alloy, and Ti6Al4V alloy.

The stress-strain hysteresis of NiTi is similar to that of bone (Fig. 11), which makes it very applicable for biomedical applications. As presented in Fig. 11, the biomechanical behaviour of NiTi is different from other biomaterials. The unique similarities between the biomechanical properties of bone and NiTi simulates the analogous behaviour of natural bone under loading-unloading conditions. The excellent biomechanical compatibility of NiTi load-baring implants prevents stress shielding effect [16], which is one of the major causes of implant failure. The maximum stress in femoral load-bearing hip NiTi implants ranges from 400 MPa to 500 MPa [50], which can be fully recovered without any residual plastic deformation [77]. Non-loading bearing NiTi implants also benefit from the high recovery strain (Fig. 11) of NiTi implants. The deformation similarities between bony tissue and NiTi implants contribute to the success of using NiTi devices for biomedical applications (Fig. 10), [77]. The precise geometrical accuracy versatility of LPBF could also be used to produce porous NiTi biomedical objects such as hip prostheses, which effectively reduce stress shielding and promote osteointegration [120].

Another major application of LPBF NiTi alloy is the production of medical stents (scaffolds) owing to the shape memory effect of the alloy and the outstanding manufacturing capability of the LPBF process. Stents are normally used to prevent the constriction of the esophagus and bile duct by keeping the anatomical structure open for the flow of fluids [121]. The stent supports the inner wall of arteries due to its intrinsic expanding capability; they could be curled at low temperatures to a small size, which permits their transportability to the targeted location. At the target location, the environmental stimuli will cause the stent to expand to prevent constriction of arteries, due to the shape memory and superelastic effects of the alloy [1]. The current maturity of the LPBF process provides freedom in stent design. Stents are now designed with unique proximal and distal sizes of vessels with new shapes and cross-branches. The LPBF process has greatly been used to produce customized stents with enhanced vascular compliance that would improve the quality of life of patients [4]. NiTi alloys are able to handle the high mechanical stress that is normally exerted on stents planted in peripheral arteries (stress during binding of the knee or walking), due to their superelasticity and stress hysteresis properties. The kink-resistant properties of NiTi also enables implantation of NiTi stents in tortuous vessels. NiTi guidewires could be used to direct catheters to various places in the body due to their kink-resistant nature. They can reach difficult-to-reach places in the body by following a tortuous path since they can rotate smoothly with excellent torque-ability. NiTi tubes could be used to deliver various medical products/devices (e.g., drugs, stents, guidewires, etc.), such as catheter shafts due to their unique properties. It is reported that over 50 % of current stents globally are made from NiTi [122,123].

### 5.2 Engineering applications (automotive, aerospace, spacecraft)

The capability of SMAs to convert thermal energy into mechanical energy by crystallographic phase change, has been the main reason for their wide industrial applications. Their non-linearity property permits them to be used in vibration isolation and large recoverable strain. The hysteresis exhibited during the transformational phases permits the dissipation of energy, making them suitable for high-value (aerospace, spacecraft, automobile) engineering designs. Compared to other smart materials, SMAs offer a simplified substantial compact solution, other than standard electromechanical or hydraulic actuators, since SMA alloy such as NiTi can actuate in a fully three-dimensional coordinate. This gives the manufacturing engineer the ability to use the LPBF method [124] to produce multimaterial actuators that can bend, twist, and extend at a large actuation frequency [119]. The litterature reveals that SMAs such as NiTi were used in F-4 in 1971 [125] aircraft hydraulic tubing coupling due to their actuation properties. NiTi alloys have been used in fixed-wing aircraft, rotorcraft, spacecraft, marine propulsion systems, and automobiles [126].

It is worth mentioning that the Smart Aircraft and Marine System Projects Demonstration (SAMPSON) [127] was the major research project that paved the way for the use of SMAs such as NiTi for widespread industrial applications. The project was mandated to identify the usefulness of SMAs such as NiTi to tailor the inlet geometry and orientation of various propulsion systems [128]. The project invented SMA wire tendons that could be used to actuate hingeless ailerons to initiate spanwise wing twisting. The NiTi tendons were first used to operate hydraulic tubing coupling of a scaled-down F-18. However, the NiTi torque could not actuate at a full-scale wing because it was difficult to use the conventional methods of manufacturing to produce large-size tendons. Meanwhile, the stress state of SMAs during actuation depends on the elastic response of the actuating structure, which also depends on its size. With the current maturity of the LPBF methods [129], it is possible to produce lare NiTi tendons, which can actuate at a higher output for a stronger actuation [130]. Large SMA components such as NiTi that can produce a larger output can be used to generate about 26 700 N force that can rotate the inlet cowl and inlet lip of jet engines [125]. The SMA tendons produced by the SAMPSON project are also used to operate jet engine nozzles during take-off and landing. The high exhaust temperature generated during take-off and landing triggers the austinite phase of the SMA tendons, causing a recovery strain to open the jet engine nozzles. At high latitudes, the low temperature induces the transition of the martensitic phase causing the nozzles to close which increases the propulsion of the flight [126]. A further search of the literature reveals that the SAMPSON project was conducted in partnership with Boeing, DARPA (Defense Advanced Research Projects Agency), and NASA. The same principles of opening and closing aircraft nozzles were used to reduce noise at take-off and landing while increasing propulsion at high altitudes. This was achieved by using the actuation properties of SMAs such as NiTi to force the chevron inward and outward to regulate the noise at take-off and landing [131]. Using the principles of AM multimaterials fabrication, integrated actuation multimaterial devices could be developed for several industrial applications apart from propulsion systems [64]. SMAs are used for morphing the wing structure in aircraft [132]. SMA such as NiTi has been embedded in the wings of aerostructures to modulate the aerofoil from symmetric to optimize performance at altitudes [133]. Shape memory wires are also used to actuate wing surface vortex generators and could be embedded in the skin of an aerodynamic surface via LPBF multimaterial manufacturing strategies to reduce turbulent drag on aircraft [126]. Due to the advantages provided by AM methods that give the possibility of manufacturing large SMA 3D structures, there is intense ongoing research on optimizing the dynamic properties of aerostructure panels via the actuating properties of SMA, to flutter the structural response [134]. Smart Material Actuated Rotor Technology (SMART) Rotor project also demonstrated that SMA-actuated tabs could be embedded or installed on rotor blades for tracking aircraft [135,136]. The possibility of manufacturing large-size NiTi shape memory alloys via LPBF process, which can demonstrate large hysteresis and strong non-linearity could be used to reduce the high vibration loads placed on payloads during launch [126]. SMA such as NiTi could be used for solar sailing. Due to the shape memory properties of NiTi radiation pressure from the sum could be converted to a propellent to drive space vehicles [126]. Self-deployed solar sailing systems were created with NiTi alloys to demonstrate self-propelling capabilities of such sail. The exact distance of the sail from the sum that can initiate complete activation of the self-propelling sail was determined by Boschetto et al. [137]. SMA have been used in many electronic devices. They can serve as prototypal linear actuators that can be used to open and close devices just by responding to positioning and displacement of corresponding SMA springs [138].

## 6 Challenges and the way forward

LPBF manufacturing of conventional alloys (Ti6Al4V, steel) is less challenging compared to LPBF manufacturing of NiTi, due to its spring-back nature, burr formation, and adhesion [3,24]. It is required that the NiTi-built components are of high density to ensure good mechanical properties, good shape memory effect, superelastic properties, suitable transformation temperatures, and low impurity content. The right energy input is required to control the composition of the alloys precisely to obtain all the above properties as required [66]. A change in the composition of the alloy could change all the desired properties drastically and impurity content above the threshold value could render the alloy not suitable for most industrial applications [139]. For example, based on the ASTM F2063-05 standard [91,92] the maximum allowable level of impurity for biomedical applications is 500 ppm. It is challenging to find the combination of optimum process parameters to produce the alloy with high density, the right chemical composition, low impurity content, and suitable shape memory properties. As already indicated by Haberland [65], the density of NiTi alloy produced by the LPBF process increases with increasing energy density as well as an increase in impurity pick up. The increase in the impurity associated with increasing laser energy density could be ascribed to the high molten pool temperature at high input energy. The high energy input produces a large molten pool with a low rate of solidification. Hence, a significant amount of impurities could be picked up in the built chamber before the NiTi molten pool solidifies [140]. This is a difficult conundrum to solve, because as high energy input is required to produce dense NiTi 3D structures, the high energy input could also lead to an increase in impurity, which renders the alloy not suitable for biomedical and other industrial applications. *Further research is required to resolve this challenge.*

Currently, no consensus-clear guidelines are in place to obtain NiTi components with high density, suitable phase transformation temperatures, and low impurity levels. This could be due to the different requirements (*chamber conditions, laser type, powder properties, etc.*) of obtaining optimum process parameters for each type of LPBF machined. Not having an established clear guideline, and the available 3D model software are also not sufficient to predict the functional and mechanical properties of the final built NiTi components making it difficult to know the result that could be obtained from each set of combinations of the process parameters. The mainstream 3D model software - AMF (additive manufacturing format), OBJ (object file format), STL (*standard tesselation language*) can only provide information about the geometry of a built component [141]. Other advanced emerging current software - 3 MF (3D manufacturing), FAV (fabricatable voxel), SVX (simple voxels) can provide information about the geometry and the properties of the material but are not capable of performing a solid modelling tasks with higher-orders [46]. The required software must provide information on the geometry, corresponding material properties, process parameters, resultant effect of the process parameters on the NiTi alloys, fictional properties, etc. The next-generation software should be able to perform all the thermodynamic calculations of different materials (multimaterial manufacturing) and provide information that can ensure the manufacturing of NiTi-multimaterial 3D structures, where the NiTi alloy could be embedded in the final component on the powder bed in one manufacturing cycle.

The resultant effect of the interrelationship of the process parameters on the functional properties and mechanical properties of the alloy is not yet well understood. A lot of research is ongoing to understand the resultant effect of the LPBF process parameters on the alloy. A clear understanding of the interrelationship of the process parameters will pave the way to precisely know which laser power, scanning, hatch distance, and powder layer thickness to select and obtain a tailored functional and mechanical property for a particular application.

Another challenge is the anisotropic characteristics of the NiTi LPBF samples which is even more difficult to control when different build orientations and scanning strategies are applied [107,142]. The build orientation significantly affects the crystallographic orientation of grains that govern the austenite and martensite phase transformation. For the determination of concise repeatable optimum process parameters, it is important to understand the exact effect of each of the build orientations on the phase transformation and the mechanical properties. A thorough survey of the literature reveals that not much attention has been focused on the effect of build orientation and scanning strategies on the phase transformation and mechanical properties of the LPBF NiTi alloy.

One of the main challenges of using NiTi SMA for industrial applications is the rate of heat transfer into and out of a SMA component. The response of the alloy is a function of the environmental stimuli which is dependent on the heat capacity and density of the alloy. As a metal, its high density and capacity can affect the repetition of actuations. Inefficient heat transfer could affect actuation frequencies. Despite the instantaneous diffusion less transformation of the martensite and austinite phases, the process of changing the temperature to drive the transformation is a function of time, which could delay the actuation and frequency. Secondly, the process of removing the heat from the SMA components via heat conduction or convection can limit the activation frequency. Several heat conduction methods have been proposed [5,143].

It is also very important to determine the exact fatigue life of NiTi alloys for each type of application. Repeated deformation at a certain magnitude would lead to failure. Most NiTi tendons could be used for multiple actuation cycles ∼10 000 cycles before a sufficient magnitude of deformation can occur [144]. As the technology of manufacturing the NiTi alloy is maturing, it is reported that NiTi alloys can undergo 600 000 cycles with consistent actuation without losing their shape memory recovery [126].

The numerous advantages of NiTi memory alloys outweigh any disadvantaged. With the emergence of AM technologies, which can produce the alloy with improved homogeneity and intricate geometrical configurations, it could be predicted that the NiTi industry would only continue to expand, and more advanced products would be developed. The SMAs global market was valued at USD 11 billion in 2021 and is projected to grow up to USD 18 billion by 2026, and USD 44 billion by 2030 at a growth rate of 14% per year [122,123].

The continuous growth in the knowledge and infrastructure of producing the NiTi alloy by LPBF would increase the quality and consistency of manufacturing NiTi 3D structures. With the advent of 4D printing and the capability of LPBF of manufacturing multimaterials continue to mature, it is expected that novel NiTi alloy would emerge with advanced capability for specific industrial applications.

## 7 Conclusion

Despite the overwhelming manufacturing capabilities demonstrated by the LPBF manufacturing process of producing intricate NiTi structures, which have the potential to revolutionize the application of SMA in various industries, a significant volume of research work is required to overcome the current challenges. The layer-wise manufacturing process could lead to internal defects (cracks) due to the high melting and cooling rates, thermal stress accumulation, anisotropic microstructure characteristics. The current focus is to establish optimized processing parameters, and comprehensively understand the effects of the various process parameters on the microstructure and the shape memory properties. Going forward it is important to conduct the following research to improve the manufacturing process and the efficiency of the alloy:

➢ Strategy to produce NiTi components with the required shape memory and superelasticity properties without post-heat treatment to reduce the production cycle, cost, and time.
➢ Determination of the effect of the anisotropy nature of the microstructure on the fatigue behaviour of the alloy especially for multiple actuation.
➢ Upgrading the simulation software to give accurate information regarding geometry, corresponding material properties, process parameters, functional properties, mechanical properties, etc. of the built LPBF NiTi components.
➢ An in-depth study on the interrelationship between the process parameters and their resultant effect on the manufacturing process to control the functional and mechanical properties of the alloy.

For the LPBF process of manufacturing NiTi alloy to attain complete maturity, it is important to establish a universal combination of process parameters that could be used to produce the alloy with desired properties for each specific industrial application.

## Data availability

The raw/processed data required to reproduce these findings can be shared on request.

## CRediT authorship contribution statement

**Thywill Cephas Dzogbewu:** Conceptualization, Data curation, Formal analysis, Funding acquisition, Investigation, Methodology, Project administration, Resources, Software, Supervision, Validation, Visualization, Writing - original draft, Writing - review & editing. **Deon Johan de Beer:** Conceptualization, Data curation, Formal analysis, Funding acquisition, Investigation, Methodology, Project administration, Resources, Software, Supervision, Validation, Visualization, Writing - original draft, Writing - review & editing.

## Declaration of competing interest

The authors declare that they have no known competing financial interests or personal relationships that could have appeared to influence the work reported in this paper.
